# Mr. Lawson Tait and Vivisection

**Published:** 1893-03-04

**Authors:** M. Armand Ruffer

**Affiliations:** 19, Iddesleigh Mansions, Westminster, S.W.


### Editor's Letter-Box.

[Our correspondents are reminded that prolixity is a great bar to publication, and that brevity of style and conciseness of statement greatly facilitate early insertion.]

### MR. LAWSON TAIT AND VIVISECTION.

Sir,—I notice in your last number a letter from Mr. Lawson Tait, F.R.C.S., in which that gentleman states that he is not an irreconcilable opponent of vivisection. If this is Mr. Tait's present position, it would be interesting to know why Mr. Tait is an honorary member of the Society for the Total Abolition of Vivisection? why he allowed his name to be paraded on the back of sandwichmen for days up and down Regent Street as the champion of anti-vivisection? and why he undertook the defence of that society at their white-washing meeting at St. James's Hall in October last? Lastly, Mr. Tait had not the courage to deny that he had been the champion of anti-vivisection when, at a medical meeting to which you allude, Dr. Barling congratulated him on his recantation, and he took care to leave the room before Mr. Horsley had the opportunity of telling him what he had to say about Mr. Tait's conduct in the past. Although Mr. Tait states that he is no longer an anti-vivisectionist, he does not appear to have forgotten their methods.—I am, Sir, your obedient servant, M. Armand Ruffer, M.D.

19, Iddesleigh Mansions, Westminster, S.W.,

February 27th, 1893.

